# Pharmacological strategies to manage oxidative stress in Huntington’s disease: from preclinical models to clinical trials

**DOI:** 10.3389/fphar.2026.1984341

**Published:** 2026-10-30

**Authors:** Sylwester Dumała, Agata Jedrzejewska, Magdalena Kwaśniak‐Butowska, Paul A. De Sousa, Marta Tomczyk

**Affiliations:** 1 Department of Biochemistry, Medical University of Gdansk, Gdansk, Poland; 2 Department of Neurology, St. Adalbert Hospital, Gdansk, Poland; 3 Department of Neurology, Neurodegenerative Disorders and Neuroimmunology, Faculty of Health Sciences, Medical University of Gdansk, Gdansk, Poland; 4 Centre for Clinical Brain Sciences, University of Edinburgh, Edinburgh, United Kingdom

**Keywords:** antioxidant therapy, clinical trials, drug repurposing, Huntington disease, mitochondrial dysfunction, oxidative stress

## Abstract

Huntington disease (HD) is a progressive neurodegenerative disorder for which effective disease-modifying treatments remain limited. Growing evidence indicates that oxidative stress, mitochondrial dysfunction, and impaired cellular antioxidant responses are important contributors to HD pathogenesis and may interact with mechanisms that drive disease progression, including somatic CAG repeat expansion. This narrative review examines therapeutic strategies aimed at restoring redox balance in HD, with particular emphasis on approaches targeting mitochondrial function, antioxidant defense, glutathione metabolism, lipid oxidation, and metal homeostasis. Although a broad range of compounds has shown beneficial effects in experimental models, translation into clinical benefit has so far been limited. This gap may reflect insufficient target engagement, inadequate brain exposure, intervention at advanced disease stages, and the difficulty of capturing biologically meaningful effects using conventional clinical endpoints. Future therapeutic development may therefore benefit from earlier intervention, biomarker-guided patient stratification, combination strategies integrating redox modulation with huntingtin-lowering approaches, and the repurposing of clinically established drugs targeting mitochondrial and redox pathways.

## 1 Introduction

Huntington disease (HD) is a monogenic, autosomal dominant neurodegenerative condition arising from a trinucleotide (CAG) expansion within exon 1 of *HTT*, the gene encoding huntingtin (MacDonald et al., 1993). Pathogenic alleles carry 36 or more repeats. Penetrance is reduced for alleles with 36–39 repeats, whereas alleles with 40 or more repeats are fully penetrant and result in the development of the disease phenotype. The CAG repeat length correlates inversely with age at symptom onset (Andrew et al., 1993; Duyao et al., 1993). Global prevalence is approximately five cases per 100,000, with populations of European ancestry showing markedly higher rates than East Asian populations, a disparity attributed largely to differences in huntingtin gene haplotypes (Pringsheim et al., 2012; Kay et al., 2017). Progression from first motor signs to death typically spans 15–20 years, and no pharmacotherapy has yet been shown to alter that trajectory (Roos, 2010).

Huntingtin (∼350 kDa) is a widely expressed protein with scaffolding and regulatory functions in vesicular trafficking, transcription, autophagy, and mitochondrial homeostasis (Kaltenbach et al., 2007; Shirasaki et al., 2012). Elongation of the amino-terminal polyglutamine tract destabilizes the native conformation and biases folding toward β-sheet-rich, aggregation-competent species (Perutz et al., 1994). The resulting cellular pathology reflects both a gain of toxic activity and partial compromise of endogenous huntingtin function. Medium spiny projection neurons of the striatum show early and disproportionate vulnerability alongside prominent microglial and astrocytic reactivity. Clinically, HD is characterized by motor, cognitive, and psychiatric manifestations. Motor features commonly include chorea, dystonia, bradykinesia, and impaired coordination. These manifestations progressively contribute to loss of functional independence. However, HD is now recognized as a multisystem condition rather than a strictly cerebral disorder: skeletal muscle wasting, cardiomyopathy, altered glucose handling, and immune dysregulation are all documented in patients and animal models (van der Burg et al., 2009; Carroll et al., 2015).

Among the downstream consequences of mutant huntingtin (mHTT), disruption of mitochondrial bioenergetics and redox homeostasis has emerged as a target for pharmacological intervention (Figure 1). HD models show abnormalities in mitochondrial electron transport chain (ETC) activity and oxidative phosphorylation (OXPHOS), reduced peroxisome proliferator-activated receptor gamma coactivator 1-alpha (PGC-1α)-dependent transcription, and impaired nuclear factor erythroid 2-related factor 2 (NRF2)-mediated antioxidant responses (Browne et al., 1997; Tabrizi et al., 1999; Cui et al., 2006; Weydt et al., 2006; Jin et al., 2013). These abnormalities can increase ROS and RNS generation while impairing cellular antioxidant capacity, promoting oxidative damage to lipids, proteins, and DNA. Experimental studies suggest that oxidative DNA lesions may influence repeat instability through DNA-repair-associated mechanisms, including OGG1-dependent base-excision repair (Kovtun et al., 2007; Budworth et al., 2015). In contrast, human genetic evidence primarily implicates mismatch-repair genes, including *MSH3*, *MLH1* and *MLH3*, together with *FAN1*, as major modifiers of somatic CAG expansion in HD (Donaldson et al., 2026). Redox-related abnormalities have also been reported in peripheral biofluids and cerebrospinal fluid during premanifest disease, although their consistency and value as therapeutic biomarkers remain uncertain (Chen et al., 2007; Klepac et al., 2007).

**FIGURE 1:**
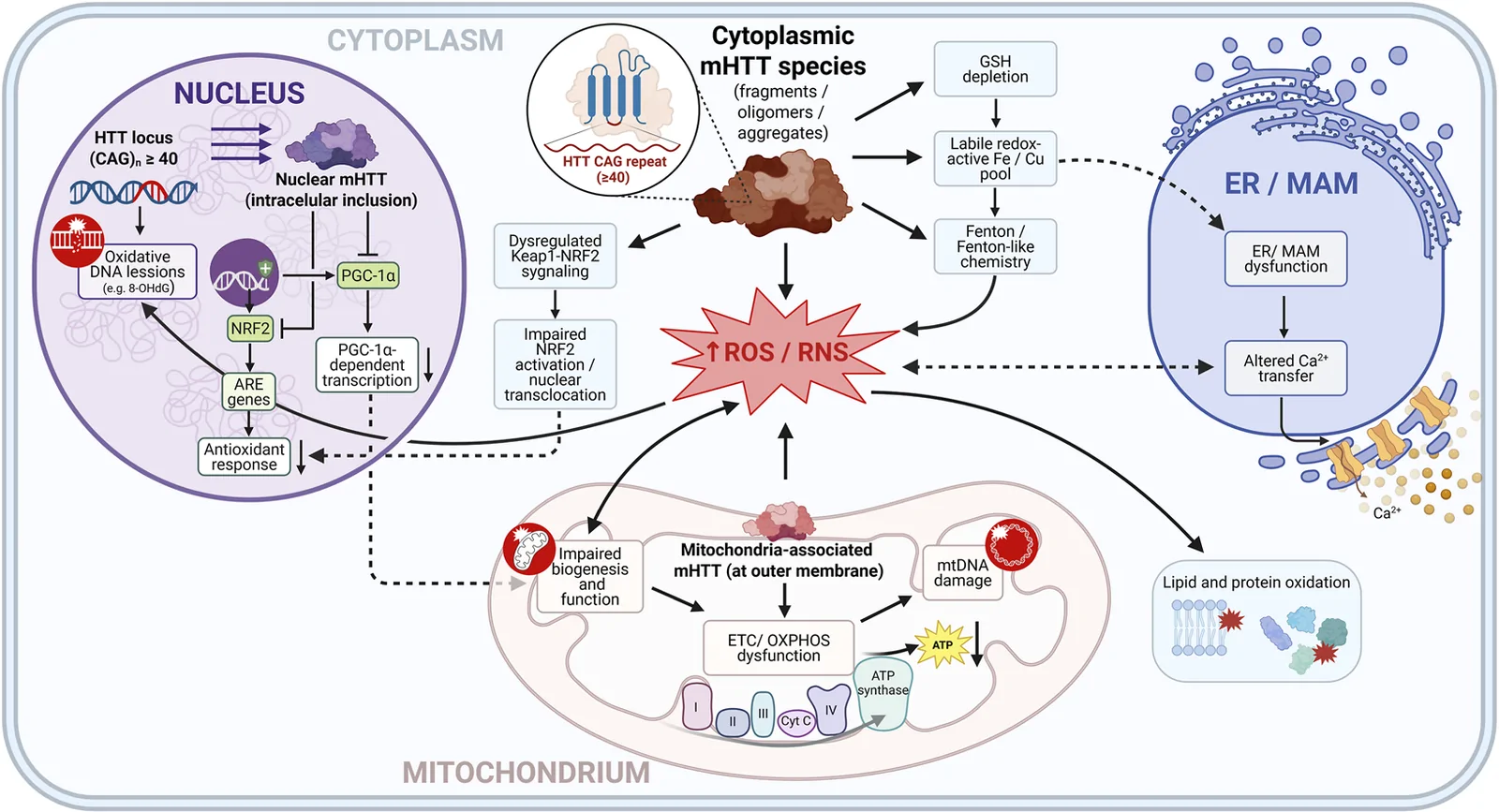
Oxidative stress in Huntington disease: cellular spatial organization of the pathogenic cascade. Mutant huntingtin (mHTT) exerts pleiotropic effects across nuclear, cytoplasmic, mitochondrial, and endoplasmic reticulum–mitochondrial compartments. Within the nucleus, mHTT interferes with the activity of key regulators of mitochondrial and redox homeostasis, including peroxisome proliferator-activated receptor gamma coactivator 1-alpha (PGC-1α), which regulates mitochondrial biogenesis and metabolic gene expression, and nuclear factor erythroid 2-related factor 2 (NRF2), which controls antioxidant response element (ARE)-driven expression of antioxidant and cytoprotective genes. In the cytoplasm, under basal conditions, Kelch-like ECH-associated protein 1 (Keap1) promotes NRF2 ubiquitination and degradation. Oxidative or electrophilic stress stabilizes NRF2 and enables its nuclear accumulation; this inducible response has been reported to be impaired in HD models. mHTT associates with mitochondrial membranes and perturbs mitochondrial proteostasis, dynamics, and bioenergetics. Electron transport chain (ETC)/oxidative phosphorylation (OXPHOS) dysfunction can increase electron leak and reactive oxygen species (ROS) generation. The endoplasmic reticulum (ER) forms close contacts with the outer mitochondrial membrane at mitochondria-associated membrane (MAM) sites, which participate in calcium exchange, mitochondrial dynamics, and lipid transfer and have been implicated in mHTT-related dysregulation. Cytoplasmic mHTT species, including soluble/oligomeric and aggregated forms, have been associated with impaired NRF2 signaling, altered glutathione homeostasis, and disturbances in metal handling. The resulting metal dyshomeostasis can further promote ROS formation through Fenton and Fenton-like chemistry. Downstream redox imbalance promotes lipid peroxidation, protein oxidation, and oxidative damage to nuclear and mitochondrial DNA, including the formation of lesions such as 8-hydroxy-2′-deoxyguanosine (8-OHdG). *Abbreviations: ARE, antioxidant response element; ATP, adenosine triphosphate; ER, endoplasmic reticulum; ETC, electron transport chain; GSH, reduced glutathione; mHTT, mutant huntingtin protein; MAM, mitochondria-associated membrane; mtDNA, mitochondrial DNA; NRF2, nuclear factor erythroid 2-related factor 2; OXPHOS, oxidative phosphorylation; 8-OHdG, 8-hydroxy-2′-deoxyguanosine; PGC-1α, peroxisome proliferator-activated receptor gamma coactivator 1-alpha; RNS, reactive nitrogen species; ROS, reactive oxygen species.*

Existing treatment remains symptomatic. Vesicular monoamine transporter 2 (VMAT2) inhibitors reduce the uptake of monoamines, including dopamine, from the cytoplasm into synaptic vesicles, thereby decreasing vesicular monoamine storage and release. This class includes tetrabenazine, deutetrabenazine, and valbenazine. These agents are used to control choreiform movements but do not modify the course of HD, and their use may be limited by adverse effects. In some cases, antipsychotic drugs may be useful, particularly in patients with comorbid behavioral disturbances or psychosis (Marshall, 2006; Frank et al., 2016). Huntingtin-lowering programmes have also highlighted dose- and safety-related challenges. Dosing in the phase III GENERATION HD1 trial of tominersen was halted after an unfavourable benefit–risk assessment, leading to evaluation of lower-exposure regimens in the subsequent GENERATION HD2 study. Development of branaplam for HD was discontinued after sensory peripheral neuropathy emerged during clinical testing (Tabrizi et al., 2019; McColgan et al., 2023; Borowsky et al., 2026). Redox- and mitochondria-directed compounds have shown beneficial effects in multiple HD models, and several have prior clinical safety data from other indications (Upadhayay and Kumar, 2024; Jiménez-Jiménez et al., 2025). However, trials of coenzyme Q10, creatine and cysteamine for HD have not demonstrated efficacy on their primary clinical endpoints (Hersch et al., 2017; McGarry et al., 2017; Verny et al., 2017). These findings highlight the persistent gap between strong experimental rationale and successful clinical translation.

This narrative review was based on targeted searches of PubMed using combinations of terms related to Huntington disease, oxidative stress, mitochondrial dysfunction, redox homeostasis, antioxidant therapy, and the individual compounds discussed. ClinicalTrials.gov was additionally searched to identify ongoing and completed clinical studies. The search covered publications available up to August 2026. No formal language restriction was applied. Priority was given to peer-reviewed primary studies with HD-specific experimental or clinical data, supplemented where relevant by mechanistic studies and evidence from other indications providing pharmacokinetic or safety context. Unpublished observations were identified explicitly and were not treated as equivalent to peer-reviewed primary evidence. Ongoing trials were identified through ClinicalTrials.gov, and their recruitment status, planned sample size, primary endpoints, and relevant study dates were verified against the registry before manuscript finalization.

## 2 Therapeutic compound classes

To compare the interventions reviewed here, compounds were grouped according to their predominant mechanism of action within redox and mitochondrial pathways. Eight classes were considered: peroxisome proliferator-activated receptor (PPAR) agonists, NRF2/antioxidant response element (ARE) activators, quinone analogues, bioenergetic substrates, mitochondria-targeted antioxidants, glutathione and cysteine donors, lipid-membrane radical scavengers, and metal-binding agents. Inclusion was based on HD-specific evidence linking a given intervention to oxidative stress, mitochondrial dysfunction, redox regulation, or closely related metabolic pathways. Because several agents act at more than one molecular target, classification reflects the mechanism most relevant to this review. The scope therefore includes both direct redox-active interventions and agents whose effects on oxidative stress are secondary to broader bioenergetic, transcriptional, proteostatic, anti-inflammatory, or metal-regulatory mechanisms. Figure 2 shows the principal intervention points for each class and organizes the eight classes into five broader intervention domains according to their main therapeutic target within redox and mitochondrial pathways.

**FIGURE 2:**
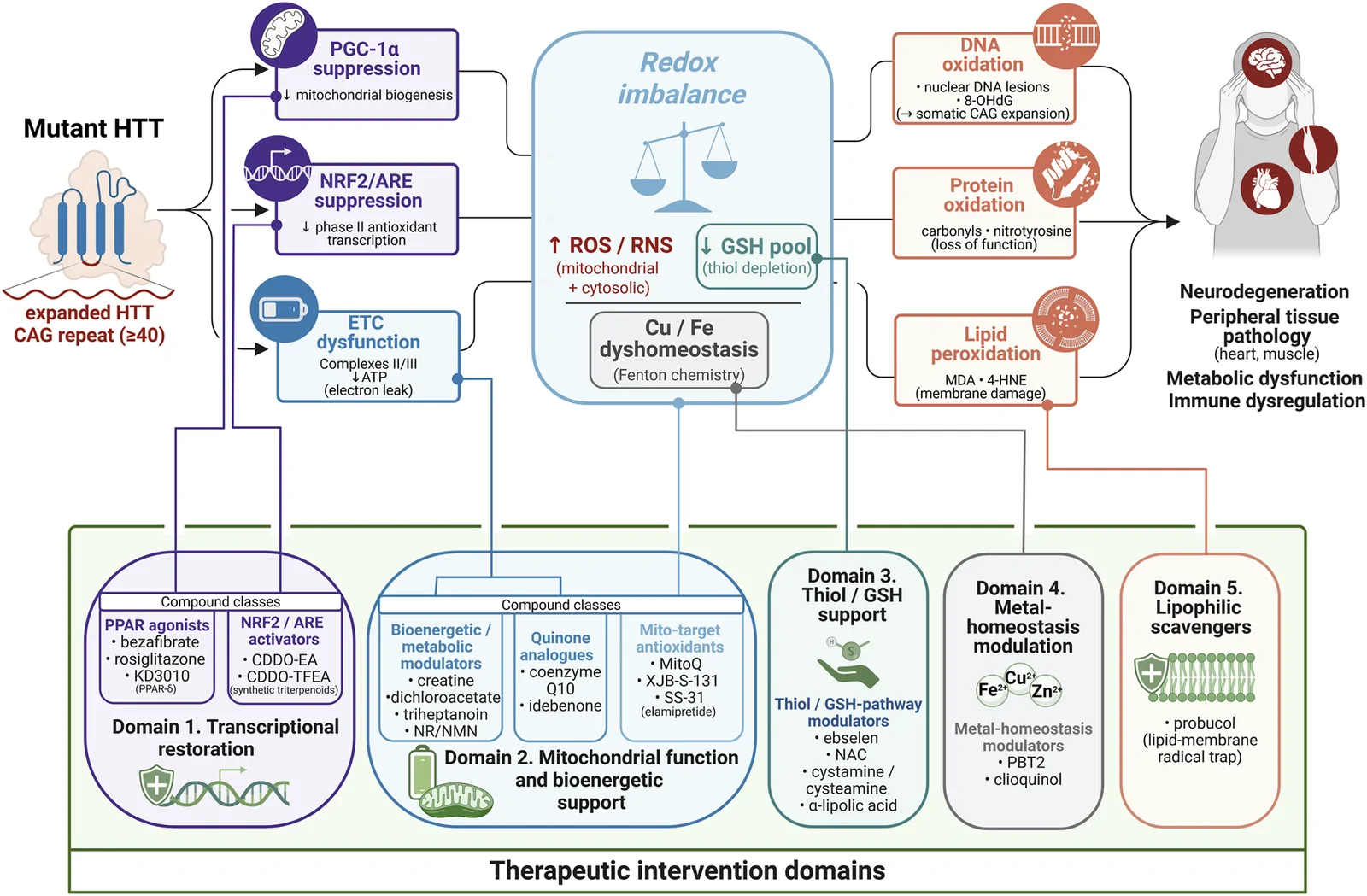
Pharmacological intervention points in redox and mitochondrial dysfunction in Huntington’s disease. mHTT-associated changes in PGC-1α-dependent transcription, NRF2 signaling, mitochondrial ETC/OXPHOS function, glutathione homeostasis and metal handling contribute to redox imbalance and oxidative damage to lipids, proteins and DNA. The eight compound classes reviewed are positioned according to their predominant mechanism of action and grouped into five broader intervention domains: transcriptional restoration, mitochondrial function and bioenergetic support, thiol/GSH support, metal-homeostasis modulation, and lipophilic radical scavenging. These domains are organizational categories rather than additional therapeutic classes.

### 2.1 PPAR agonists

PPARs are ligand-regulated transcription factors within the superfamily of nuclear receptors. The family comprises three members, PPARα, PPARβ/δ, and PPARγ, which regulate genes involved in fatty-acid and glucose metabolism, mitochondrial biogenesis, respiratory function, and inflammatory signaling. These pathways are relevant to the bioenergetic and redox abnormalities reported in HD. PPAR/PGC-1α signaling is impaired in HD and has been reported to be reduced in both CNS and peripheral tissues in experimental models (Johri et al., 2012; Tomczyk et al., 2022). Three pharmacological approaches have been used to activate this axis: PPARγ-selective agonists (glitazones), the pan-PPAR agonist bezafibrate, and PPARδ-selective agonists.

PPARγ agonists have been evaluated in several HD cell models. In immortalized striatal progenitor cells carrying the Q111 huntingtin knock-in allele, a calcium challenge with thapsigargin increased ROS production and reduced mitochondrial membrane potential. Rosiglitazone prevented both, raising mitochondrial mass. The involvement of PPARγ was supported by the loss of this protective effect in the presence of the PPARγ antagonist GW9662 (Quintanilla et al., 2008). The same study showed that γ-receptor expression and transcriptional output were already reduced in the mutant cells (Quintanilla et al., 2008). Similar effects were reported in mHTT-expressing neuroblastoma (N2A) cells. Rosiglitazone increased expression of PPARγ and downstream regulators of mitochondrial biogenesis, PGC-1α, nuclear respiratory factor 1, and mitochondrial transcription factor A, while enhancing mitochondrial biogenesis and improving cell survival. It also lowered the aggregate load, restored ubiquitin-proteasome throughput, induced the chaperones heat-shock factor 1 and HSP27/70, and normalized changes in ER-stress markers, including BiP, CHOP, and ASK1 (Chiang et al., 2015).

Protective effects have also been reported *in vivo*. Rosiglitazone, a PPARγ agonist of the thiazolidinedione class, was tested in R6/2 mice, in which PPARγ expression is markedly reduced. Mice received a 0.01% rosiglitazone-supplemented diet from 4 weeks of age for 8 weeks. Chronic treatment attenuated body-weight loss and motor decline, reduced the number of mHTT aggregates, and prolonged survival (Chiang et al., 2010). This was associated with increased expression of downstream targets, including PGC-1α, brain-derived neurotrophic factor (BDNF), and Bcl-2 (Chiang et al., 2010, 2012). Rosiglitazone was also detected in cortical tissue from treated R6/2 mice, supporting brain exposure in this model (Chiang et al., 2010).

The pan-PPAR agonist bezafibrate activates all three PPAR isoforms. In R6/2 mice, dietary bezafibrate restored PGC-1α, PPAR expression, and downstream target-gene expression toward wild-type levels, and improved phenotypic measures and survival. Within the striatum, it reduced astrogliosis and neuronal atrophy and increased mitochondrial abundance (Johri et al., 2012). Similar effects were reported in the BACHD full-length mHTT model. In BACHD mice, bezafibrate partially restored PPARγ/PPARδ/PGC-1α signaling that is impaired by mHTT expression, drove mitochondrial biogenesis, and reinforced striatal antioxidant capacity. Bezafibrate also attenuated motor deficits and striatal pathology (Chandra et al., 2016). In addition, in rats exposed to the complex II inhibitor 3-nitropropionic acid (3-NP), bezafibrate attenuated changes in antioxidant enzyme activity, glutathione status, mitochondrial markers and synaptophysin levels (Brondani et al., 2023). PPARδ has been reported to interact directly with huntingtin, whereas mHTT suppresses PPARδ transcriptional activity. In HD mouse models, the δ agonist KD3010 improved motor performance, delayed neurodegeneration, and extended survival. The same compound reduced mHTT-associated toxicity in medium spiny neuron-like cells differentiated from patient-derived induced pluripotent stem cells (iPSCs) (Dickey et al., 2016). Peripheral effects of PPAR agonists have also been reported in HD models. In R6/2 mice, bezafibrate attenuated the shift from type I to type II muscle fibres and increased mitochondrial numbers in skeletal muscle. It also reduced lipid accumulation and vacuolar changes in brown adipose tissue (Johri et al., 2012). Rosiglitazone produced similar peripheral effects in R6/1 mice. It improved grip strength, cardiac mechanical function, and indices of cardiac energy metabolism (increased the adenine-nucleotide pool, glucose oxidation, and raised citrate synthase activity as an index of mitochondrial content). Total antioxidant status was elevated, and the circulating markers of nucleotide degradation were reduced (Tomczyk et al., 2022). These findings indicate that PPAR agonism can modify peripheral phenotypes in HD models in addition to CNS-related outcomes.

PPAR agonists have not yet been evaluated clinically in HD. Bezafibrate and fenofibrate are lipid-lowering agents with predominant PPARα activity, whereas rosiglitazone and pioglitazone are PPARγ agonists developed for the treatment of type 2 diabetes. Rosiglitazone has been subject to major regulatory restrictions, including suspension of its marketing authorization in Europe because of cardiovascular safety concerns. Prior clinical use of these agents in other indications provides supportive information on human exposure and tolerability, but does not establish safety at HD-relevant doses or treatment durations, nor adequate CNS exposure, regional brain exposure, target engagement, or efficacy in patients with HD. Thus, the HD-specific evidence for PPAR agonists remains preclinical.

### 2.2 NRF2/ARE activators

NRF2 is a principal regulator of the inducible cellular antioxidant response. Under basal conditions, Kelch-like ECH-associated protein 1 (Keap1) promotes NRF2 ubiquitination and proteasomal degradation. Oxidative or electrophilic modification of Keap1 stabilizes NRF2, facilitating its nuclear accumulation and activation of ARE-dependent transcription. In striatal cells expressing full-length mHTT, the NRF2 response is dysregulated (Jin et al., 2013). Pharmacological activation of NRF2 has therefore been investigated as a strategy to enhance antioxidant capacity in HD models. Synthetic oleanane triterpenoids derived from oleanolic acid are potent activators of NRF2/ARE signaling. Their shared scaffold, 2-cyano-3,12-dioxooleana-1,9-dien-28-oic acid (CDDO), contains electrophilic enone moieties that can covalently modify cysteine residues in Keap1. This electrophilic reactivity contributes to NRF2 activation and has also been associated with anti-inflammatory effects (Dinkova-Kostova et al., 2005).

Two CDDO derivatives have been evaluated in an HD mouse model. In N171-82Q transgenic mice, CDDO-ethyl amide (CDDO-EA) and CDDO-trifluoroethyl amide (CDDO-TFEA) were supplied in the feed at a range of concentrations from postnatal day 30 (Stack et al., 2010). Both increased expression of ARE-regulated genes in brain and peripheral tissues, reduced markers of oxidative damage, improved motor performance, and prolonged survival. Each also reduced striatal atrophy and reversed brown-adipose-tissue vacuolation (Stack et al., 2010). The induction of ARE-controlled genes in brain tissue provides evidence of central pharmacodynamic activity in this model.

HD-specific evidence for CDDO-EA and CDDO-TFEA is currently derived primarily from *in vivo* studies, as we did not identify corresponding *in vitro* studies in HD models. These compounds have not been evaluated clinically in HD. Thus, the HD-specific evidence remains preclinical, and CNS exposure, regional brain exposure, biochemical target engagement, and the safety of sustained NRF2 activation in patients remain unknown.

### 2.3 Quinone analogues

The quinone analogues share a redox-active benzoquinone head that cycles between oxidised and reduced states, which enables electron transfer and, in the reduced state, antioxidant activity within lipid membranes. Two members have featured in HD research: coenzyme Q10 (CoQ10), the endogenous ubiquinone that transfers electrons from complexes I and II to complex III, and idebenone, a synthetic short-chain benzoquinone with greater hydrophilicity than CoQ10. Because CoQ10 is an essential component of the mitochondrial ETC and contributes to limiting electron leakage and ROS formation, its supplementation has been proposed as a potential therapeutic strategy in HD, where mitochondrial bioenergetics dysfunction and redox imbalance are prominent features.

Direct evidence from HD-specific cell models is limited; most *in vitro* support comes from neuronal lines placed under generic oxidative or metabolic stress, where CoQ10 lowers ROS output and preserves mitochondrial membrane potential, with greater effects reported for more readily dispersed formulations (Somayajulu et al., 2005). These findings provide mechanistic support for antioxidant and mitochondrial effects but do not constitute HD-specific cellular evidence.

In CAG140 mice fed CoQ10 in chow, the lower 0.2% dose improved early behavioural deficits and corrected part of the striatal transcriptional signature, while the higher 0.6% dose did less. However, the behavioural and transcriptional effects occurred without a detectable reduction in huntingtin aggregate burden (Hickey et al., 2012). Thus, the HD-specific preclinical evidence for CoQ10 is derived primarily from an *in vivo* genetic model that is consistent with downstream functional and transcriptional effects rather than a direct effect on mHTT aggregation.

CoQ10 has been clinically tested in HD more thoroughly than any other antioxidant, in several clinical trials using progressively higher doses. An early 6-month open-label study in ten patients found that CoQ10 at 600–1,200 mg/day was well tolerated, but the uncontrolled design and small sample precluded conclusions about efficacy (Feigin et al., 1996). The multicentre CARE-HD trial randomised 347 patients with early-stage HD to CoQ10 (600 mg/day), remacemide, both treatments, or placebo for 30 months. None of the active treatments significantly reduced decline in Total Functional Capacity (TFC), the primary outcome (Huntington Study Group, 2001). Subsequent dose-escalation studies evaluated doses up to 3,600 mg/day, while 2,400 mg/day was selected for further clinical evaluation on the basis of systemic exposure and tolerability (Hyson et al., 2010). That dose was carried into the definitive 2CARE trial, which followed 609 early-stage patients on 2,400 mg/day for up to 5 years before stopping early for futility; no group difference emerged on TFC or any secondary measure, and the investigators concluded that CoQ10 does not slow the course of HD (McGarry et al., 2017). Thus, despite preclinical activity and adequate systemic exposure at high oral doses, CoQ10 has not demonstrated clinical efficacy in HD. Limited CNS exposure and the absence of demonstrated striatal target engagement could explain the negative clinical findings with CoQ10, although these mechanisms have not been established as the cause of treatment failure. Whether treatment initiation after symptom onset may also have contributed to the lack of efficacy remains uncertain.

Idebenone was developed as a shorter-chain analogue of CoQ10 with different pharmacokinetic properties. A short isoprenoid tail gives it better water solubility and tissue distribution, and in principle it can transfer electrons to complex III even when complex I is compromised. However, its antioxidant activity is dependent on two-electron reduction to idebenol, and without that reduction it can form an unstable semiquinone that acts as a pro-oxidant. This reduction depends largely on NAD(P)H:quinone oxidoreductase 1 (NQO1) (Jaber and Polster, 2015). This dependence may limit its activity in neurons, because NQO1 is expressed mainly by glia and is scarce in neurons. Tested directly, idebenone supported respiration and bypassed a complex I block in cortical astrocytes but lowered respiratory capacity in neurons. Delivering NQO1 into neurons restored the bypass, confirming that idebenone acts as an NQO1-dependent prodrug (Jaber et al., 2020). These findings provide mechanistic *in vitro* evidence for cell-type-dependent idebenone bioactivation, but do not establish CNS target engagement in HD.

Clinical evidence in HD has not demonstrated efficacy. In a trial of 100 patients with HD randomised to idebenone (90 mg three times daily; 270 mg/day) or placebo for 12 months, there was no significant difference between groups in the two primary endpoints, the HD Activities of Daily Living scale (ADL) and the Quantified Neurologic Examination (QNE) (Ranen et al., 1996). The study was underpowered to detect anything short of a near-complete arrest of disease progression. These findings suggest that neuronal NQO1 availability may influence idebenone activity, although this has not been tested therapeutically in HD. Thus, the proposed limitation related to neuronal bioactivation remains mechanistically plausible but has not been demonstrated as the reason for the negative clinical result. Human exposure in this trial confirms clinical administration but does not by itself establish adequate regional CNS exposure or biochemical target engagement.

Neither CoQ10 nor idebenone has demonstrated clinical benefit in HD. Limited CNS exposure and, for idebenone, cell-type-dependent bioactivation have been proposed as potential limitations, although the available data do not establish these as the causes of clinical failure. Overall, this class illustrates the distinction between systemic exposure, CNS delivery, target engagement, downstream pharmacodynamic effects, and clinical efficacy: evidence at one level does not establish evidence at the next.

### 2.4 Bioenergetic substrates

These compounds primarily target cellular energy metabolism rather than directly scavenging reactive species or activating antioxidant transcription. Because bioenergetic abnormalities are well documented in HD, interventions that support phosphagen buffering, oxidative substrate utilization, anaplerosis, or nicotinamide adenine dinucleotide (NAD^+^) metabolism have been investigated as potential therapeutic strategies. Four approaches are considered here: creatine, dichloroacetate (DCA), triheptanoin, and NAD^+^ precursors. The stage of evidence differs substantially across these approaches, ranging from predominantly preclinical evidence for DCA and NAD^+^ precursors to direct clinical evaluation of creatine and triheptanoin in patients with HD.

Creatine contributes to short-term energy buffering in tissues with high and fluctuating adenosine triphosphate (ATP) demand, including brain and skeletal muscle. Through the creatine kinase/phosphocreatine system, it facilitates rapid regeneration of ATP from adenosine diphosphate (ADP). Creatine has also been reported to exert antioxidant effects that may be partly independent of its role in energy buffering.

*In vitro* studies support both effects. In cultured striatal and hippocampal neurons, phosphocreatine and creatine attenuated toxicity induced by the complex II inhibitor 3-NP and, when administered before glutamate exposure, provided protection comparable to that of the non-competitive NMDA receptor antagonist MK-801 (Brustovetsky et al., 2001). In human neuroblastoma cells, creatine reduced ROS and nitric oxide generation provoked by glutamate or hydrogen peroxide and lowered the nitrite formed from a nitric-oxide donor, supporting a possible direct scavenging effect rather than an indirect metabolic effect (Cunha et al., 2016). That scavenging was confirmed in non-neuronal lines, where creatine spared the intracellular thiol pool from oxidant consumption and left a mass-spectrometry-detectable oxidation by-product, evidence of a chemical reaction with the radical rather than enzyme induction (Sestili et al., 2006). Creatine has also been linked to mitochondrial quality control: in oxidatively stressed myoblasts it activated the energy sensor AMP-activated protein kinase (AMPK) and raised PGC-1α, preserving mitochondrial structure and function (Barbieri et al., 2016).

*In vivo*, creatine has also been evaluated in combination with CoQ10 in R6/2 mice. Each treatment increased median survival, whereas combined treatment resulted in greater improvement in rotarod performance. The combination also reduced striatal lipid peroxidation and DNA oxidation and preserved GSH-related measures following 3-NP exposure (Yang et al., 2009). These findings provide preclinical evidence of downstream metabolic and redox effects but do not establish biochemical target engagement.

Creatine has the longest clinical record of the four bioenergetics substrates. A pilot study in 41 patients with stage I–III HD evaluated creatine at 5 g/day for 1 year and found no significant change in UHDRS motor score, muscle strength, peak oxygen uptake, or cognition (Verbessem et al., 2003). The PRECREST study then took high-dose creatine, 30 g/day, into premanifest carriers and people at 50% genetic risk, across a 6-month blinded phase and a year-long open-label extension. High-dose creatine was generally tolerated during the study period. Moreover, the study identified differences in longitudinal MRI measures, providing an exploratory imaging signal in the premanifest setting (Rosas et al., 2014). These MRI findings should be interpreted as exploratory downstream imaging effects rather than evidence of biochemical target engagement or clinical efficacy. The definitive test, CREST-E, escalated creatine dosing to 40 g/day in 553 early-stage patients across multiple sites. Functional decline was numerically greater with creatine than with placebo (0.82 vs 0.70 TFC points/year), and the trial was terminated for futility (Hersch et al., 2017). Differences between PRECREST and CREST-E do not establish a stage-dependent treatment effect, because the studies differed substantially in population, design, duration, and outcome measures. Overall, despite extensive clinical evaluation, creatine has not demonstrated clinical efficacy in symptomatic HD.

DCA inhibits pyruvate dehydrogenase kinase (PDK), thereby reducing inhibitory phosphorylation of the pyruvate dehydrogenase complex (PDHC) and promoting oxidative conversion of pyruvate to acetyl-CoA. HD striatal cell models show reduced PDHC activity, increased phosphorylation of the E1α subunit, and increased expression of PDK1 and PDK3. DCA increased mitochondrial respiration and reduced ROS production in these cells (Naia et al., 2017). *In vivo*, DCA has been evaluated in two transgenic HD mouse lines, R6/2 and N171-82Q. DCA lengthened survival, improved motor performance, slowed weight loss, reduced striatal neuronal atrophy, and prevented the diabetic phenotype. The fall in active PDHC seen in R6/2 mice was corrected by treatment (Andreassen et al., 2001b). DCA has not been evaluated clinically in HD. Previous clinical use in disorders associated with lactic acidosis provides information on human exposure and tolerability, but does not establish safety at HD-relevant doses or treatment durations, adequate CNS or regional brain exposure, biochemical target engagement, or efficacy in HD. Chronic exposure can cause peripheral neuropathy, which is an important limitation for long-term treatment in HD. Thus, the HD-specific evidence for DCA remains preclinical.

Triheptanoin is an odd-chain triglyceride that generates both acetyl-CoA and propionyl-CoA; the latter is converted to succinyl-CoA and thereby contributes anaplerotic carbon to the TCA cycle. Its five-carbon ketone bodies have been reported to cross the BBB, delivering anaplerotic carbon to the brain (Mochel, 2017). However, BBB passage does not by itself establish regional CNS exposure or biochemical target engagement in HD.

HD-specific preclinical evidence for triheptanoin is limited, and its evaluation in HD has proceeded primarily through clinical metabolic and imaging studies. A short study in six patients showed that triheptanoin, administered at a dietary dose of 1 g/kg body weight per day, was generally well tolerated during the study period, normalized muscle pH control in the two participants who began with exercise-induced acidosis, and increased serum insulin-like growth factor 1 (Mochel et al., 2010). A subsequent open-label study assessed cerebral bioenergetics using phosphorus-31 magnetic resonance spectroscopy (^31^P-MRS) in ten early-stage patients and found an abnormal energy profile at rest that a month of triheptanoin corrected, restoring the normal rise in the inorganic-phosphate-to-phosphocreatine ratio during visual stimulation (Adanyeguh et al., 2015). The phase II trial that followed was larger and blinded: 107 early-stage patients received triheptanoin or placebo for 6 months. The prespecified primary endpoint showed no significant difference in caudate atrophy between triheptanoin and placebo. A subsequent *post hoc* comparison with an external placebo cohort suggested less caudate atrophy after 12 months of treatment, but this analysis cannot establish efficacy (Mochel et al., 2025). Thus, triheptanoin has undergone direct clinical evaluation in HD, but clinical efficacy has not been established.

The final strategy considered in this class targets NAD^+^ metabolism rather than providing a direct energetic substrate. NAD^+^ plays a central role in cellular energy metabolism, serving as a redox cofactor in multiple metabolic reactions and as a substrate for NAD^+^-consuming enzymes, including sirtuins, poly(ADP-ribose) polymerases (PARPs), and CD38. Alterations in NAD^+^ homeostasis have been reported in HD. In particular, oxidative DNA damage can promote PARP activation and thereby increase NAD^+^ utilization, providing a mechanistic link between disturbed NAD^+^ metabolism and the DNA damage–oxidative stress axis described above. Accordingly, restoration of NAD^+^ availability through supplementation with salvage-pathway precursors, including nicotinamide, nicotinamide riboside (NR), and nicotinamide mononucleotide (NMN), has emerged as a potential therapeutic strategy and has shown beneficial effects in preclinical models of several neurodegenerative disorders (Reiten et al., 2021). Among NAD^+^ precursors, the most clearly documented peer-reviewed evidence in HD is available for nicotinamide. In R6/1 mice, nicotinamide treatment improved motor performance and increased both mRNA and protein expression of BDNF and PGC-1α, the latter being consistent with enhanced transcriptional regulation of mitochondrial biogenesis and oxidative metabolism. The functional and transcriptional effects occurred without a detectable reduction in mHTT aggregate burden (Hathorn et al., 2011). Interpretation of these findings is additionally complicated by the pharmacological properties of nicotinamide itself, which at sufficiently high concentrations can inhibit sirtuins; thus, its effects cannot be attributed solely to restoration of the NAD^+^ pool. Evidence for NR in HD remains less established. Effects involving sirtuin activation, PGC-1α signaling, and improvements in motor and molecular phenotypes in mutant striatal cells and two HD mouse models have been described in review literature as unpublished observations and therefore cannot yet be evaluated as primary experimental evidence (Lloret and Beal, 2019). These observations should therefore not be considered equivalent to peer-reviewed preclinical evidence. Clinical evaluation of NAD^+^-restoring strategies in HD is now beginning. NAD-HD (NCT06853743), a phase II randomized controlled study, is evaluating the safety, tolerability, biological target engagement and potential clinical effects of oral nicotinamide riboside supplementation in individuals with HD. No efficacy results are currently available; therefore, this ongoing study should not be interpreted as evidence of clinical benefit. Biological target engagement, if demonstrated, should also be distinguished from downstream pharmacodynamic effects and clinical efficacy.

Bioenergetic interventions have produced beneficial effects in several HD models, but the level of evidence differs across compounds and clinical efficacy remains unproven. CREST-E showed no benefit of high-dose creatine in symptomatic HD, and the randomized phase of the triheptanoin study did not meet its primary imaging endpoint. In contrast, DCA remains supported by HD-specific preclinical evidence only. Exploratory imaging findings from PRECREST and longer-term triheptanoin analyses warrant further study but do not establish disease modification. Ongoing studies of NAD^+^-restoring approaches will determine whether metabolic target engagement can be demonstrated in patients.

### 2.5 Mitochondria-targeted antioxidants

Several strategies have been developed to increase mitochondrial localization of redox-active compounds. One approach employs a lipophilic triphenylphosphonium (TPP^+^) cation, which accumulates within the mitochondrial matrix in response to the negative inner-membrane potential and thereby markedly increases the intramitochondrial concentration of the conjugated antioxidant. A second strategy uses mitochondria-targeting peptide sequences whose physicochemical properties promote preferential association with and uptake by mitochondria. A third approach relies on peptides with affinity for cardiolipin, a phospholipid enriched in the inner mitochondrial membrane, enabling direct localization of the compound to this membrane compartment. Mitochondrial targeting may increase local drug concentrations relative to non-targeted antioxidants such as CoQ10. Three representative mitochondria-targeted compounds have been evaluated in HD models: MitoQ, a TPP^+^-conjugated ubiquinone derivative; XJB-5–131, a mitochondria-targeted nitroxide-containing peptide conjugate; and SS-31 (elamipretide), a cardiolipin-binding tetrapeptide.

MitoQ consists of a ubiquinone moiety conjugated to TPP^+^, which promotes mitochondrial accumulation. Within mitochondria, the ubiquinone group is reduced to ubiquinol and can undergo redox cycling while limiting lipid peroxidation and other oxidative reactions (Murphy and Smith, 2007). *In vitro*, in striatal cells carrying the homozygous Q111 knock-in allele, MitoQ reduced ROS production and lipid peroxidation, raised ATP, and increased transcription of the biogenesis programme (PGC-1α, NRF1, NRF2, TFAM), ETC subunits, and the synaptic markers synaptophysin and PSD95. Collectively these findings suggested recovery of both mitochondrial and synaptic function (Yin et al., 2016). Complementary experiments with MitoParaquat (MitoPQ), a TPP^+^-targeted redox cycler that increases mitochondrial superoxide, showed increased mHTT aggregation in cultured human cells and motor abnormalities in zebrafish larvae (Pinho et al., 2019). These findings support an association between mitochondrial superoxide and mHTT-related phenotypes.

*In vivo*, in R6/2 mice, MitoQ improved fine motor performance and reduced oxidative damage, with prominent effects in skeletal muscle, where it also attenuated increased autophagy. Brain autophagy markers were unchanged, which is consistent with limited brain exposure reported in this model but does not by itself establish the extent of regional CNS exposure or target engagement (Pinho et al., 2020). The predominantly peripheral effects therefore highlight a potential CNS-delivery limitation despite mitochondrial targeting.

MitoQ has not been trialled in HD, but two controlled studies in other diseases define its human profile. It reduced serum aminotransferase concentrations in patients with hepatitis C, indicating an effect on biochemical markers of liver injury (Gane et al., 2010). In 128 newly diagnosed Parkinson’s disease patients, a year of oral MitoQ was generally well tolerated but did not slow clinical progression (Snow et al., 2010). These studies demonstrate systemic human exposure and peripheral pharmacodynamic activity, but do not establish adequate CNS exposure, biochemical target engagement, safety at HD-specific doses or treatment durations, or efficacy in HD.

XJB-5–131 combines a hemigramicidin S-derived mitochondrial-targeting moiety with a nitroxide redox-active group (Wipf et al., 2005). The nitroxide undergoes reversible redox cycling and can catalytically limit oxidative reactions (Jiang et al., 2007). Mitochondrial targeting of the hemigramicidin–nitroxide scaffold was first demonstrated in generic cellular models (Wipf et al., 2005). In primary striatal neurons derived from HD150KI mice, XJB-5-131 localized to mitochondria and improved neuronal survival (Xun et al., 2012). *In vivo*, given early to HdhQ150 knock-in mice, from 7 to 57 weeks of age, it slowed motor decline and weight loss, improved neuronal survival, cut oxidative damage to mitochondrial DNA (mtDNA), and held mtDNA copy number steady. XJB-5–131 also reduced somatic CAG expansion in HdhQ150 mice, in parallel with reduced oxidative DNA damage. However, this association does not establish that reduced oxidative damage mediates the effect on somatic expansion, and the mechanistic relationship between these changes remains uncertain (Xun et al., 2012). To date there have been no human trials of this compound. However, clinical translation may require further characterization of pharmacokinetics, CNS exposure, dosing route and long-term safety.

SS-31 (elamipretide) is a cardiolipin-binding tetrapeptide that localizes to the inner mitochondrial membrane. Its interaction with cardiolipin has been associated with improved respiratory function, ATP production and mitochondrial membrane organization, together with reduced oxidative stress (Nhu et al., 2022). HD-specific evidence is currently limited to *in vitro* studies. In homozygous Q111 striatal cells, SS-31 reduced ROS and lipid peroxidation, increased ATP levels and mitochondrial biogenesis- and synaptic-related transcripts, and improved mitochondrial function (Yin et al., 2016). Elamipretide received accelerated FDA approval in 2025 for the treatment of Barth syndrome, providing human pharmacokinetic and tolerability experience with the compound (U.S. Food and Drug Administration, 2025; Thompson et al., 2024). However, these data do not constitute clinical evidence in HD and do not establish HD-specific CNS exposure, biochemical target engagement, safety at HD-relevant doses or treatment durations, or efficacy.

Taken together, mitochondria-targeted antioxidants are designed to concentrate antioxidant activity directly within mitochondrial compartments implicated in HD-associated redox dysfunction. Among these compounds, XJB-5–131 is of particular interest because its reported effects extend beyond attenuation of oxidative damage and include reduced somatic CAG-repeat expansion in preclinical models. Whether these effects are mechanistically linked remains unclear. MitoQ has shown predominantly peripheral effects in R6/2 mice, whereas XJB-5–131 has demonstrated *in vivo* activity following parenteral administration. HD-specific evidence for elamipretide remains limited to cell models. None of these agents has been evaluated clinically in HD. A major unresolved issue is whether these compounds can achieve sufficient and sustained exposure in vulnerable CNS regions and demonstrate biochemical target engagement in HD.

### 2.6 Glutathione/cysteine donors

GSH is a major intracellular redox buffer synthesized from glutamate, cysteine and glycine. It supports peroxide detoxification primarily by serving as a reducing substrate for glutathione peroxidases. Alterations in GSH metabolism have been reported in HD models, although these do not necessarily manifest as simple GSH depletion. Striatal cells carrying full-length mHTT show increased ROS and caspase-3 activity, yet their total glutathione, in both reduced and oxidised (GSSG) forms, is increased rather than depleted, and the activities of glutathione peroxidase and reductase are also elevated (Ribeiro et al., 2012). Cysteine availability, which is required for GSH synthesis, is reduced in the HD striatum because of decreased expression of cystathionine γ-lyase (CSE), an enzyme involved in cysteine biosynthesis (Paul et al., 2014). *De novo* synthesis of GSH may also be impaired because the activities of glutamate-cysteine ligase and glutathione synthase are reduced. Reduced multidrug-resistance-associated protein 1-mediated GSH export may provide an additional explanation for intracellular GSH accumulation despite impaired GSH homeostasis (Ribeiro et al., 2012). These findings suggest that total GSH concentration alone may not adequately reflect functional antioxidant capacity and provide a rationale for targeting GSH synthesis, utilization or precursor availability. Several compounds have been investigated in this context. Ebselen acts as a glutathione peroxidase mimetic, thereby facilitating the reduction of peroxides using GSH as an electron donor. Cystamine and its reduced derivative cysteamine, as well as N-acetylcysteine (NAC), can increase cysteine availability and thereby support *de novo* glutathione synthesis. α-Lipoic acid has also been proposed to enhance cellular thiol homeostasis, in part by promoting cystine utilization and increasing the availability of substrates required for glutathione synthesis.

Ebselen is an organoselenium compound with GPx-mimetic activity that promotes peroxide reduction in the presence of GSH. Evidence in HD models originated from an unbiased genetic screen rather than a targeted hypothesis: a genome-wide search for genes that blunt mHTT toxicity in yeast returned two glutathione peroxidases among the strongest suppressors, and follow-up studies in yeast, mammalian cells, and *Drosophila* showed that increasing GPx activity reduced mHTT-associated toxicity more effectively than several other antioxidant strategies tested in parallel (Mason et al., 2013). In neuron-like PC12 cells, overexpressing GPx lowered ROS and roughly halved the apoptotic caspase signal, and ebselen reproduced the effect pharmacologically, reducing ROS by approximately two-thirds and decreasing apoptotic signalling (Mason et al., 2013). Increasing GPx activity did not impair autophagic flux in the experimental systems examined (Mason et al., 2013). Ebselen has been evaluated clinically in other indications, providing information on human exposure, pharmacokinetics, and tolerability; however, these data do not establish safety at HD-relevant doses or durations, and ebselen has not been evaluated clinically in HD.

Cystamine is a small disulfide that can increase cysteine availability and support GSH synthesis. It was first pursued for a different reason, as an inhibitor of transglutaminase (TG), an enzyme that cross-links and helps stabilise huntingtin aggregates. Protection was retained in R6/2 mice lacking transglutaminase activity, suggesting that mechanisms other than transglutaminase inhibition contribute to its effects (Bailey and Johnson, 2006). Consistent with this interpretation, cystamine raised brain L-cysteine in R6/2 mice and reduced striatal injury from 3-NP, pointing to antioxidant rather than purely anti-aggregation action (Fox et al., 2004). The cellular evidence ties the mechanism to glutathione. In mutant STHdhQ111 striatal cells, the complex II inhibitor 3-NP collapses the mitochondrial membrane potential; cystamine partially preserved mitochondrial membrane potential at 50 μM and fully at 250 μM, while cysteamine showed comparable effects across concentrations of 50–500 μM (Mao et al., 2006). Pre-treatment with either compound stopped the 3-NP-driven fall in mitochondrial and cytosolic GSH, and GSH depletion with buthionine sulfoximine abolished the protective effect, placing glutathione at the centre of the mechanism (Mao et al., 2006). In animal studies, the structural benefit was clear but incomplete. Oral cystamine extended survival and improved body weight and motor performance in R6/2 mice while normalising elevated TG markers (Dedeoglu et al., 2002). In the slower, full-length YAC128 model it prevented striatal neuron loss and reduced striatal volume loss and neuronal atrophy (Van Raamsdonk et al., 2005). The same YAC128 study also highlighted an important limitation of this approach: despite preserving striatal structure, cystamine did not correct motor dysfunction or restore DARPP-32 expression, the dopaminergic-signalling protein. Thus, preservation of striatal structure was not accompanied by comparable improvement in all functional or molecular outcomes (Van Raamsdonk et al., 2005).

The pharmacokinetic limitations of cystamine have largely restricted its use to preclinical studies, whereas its reduced form, cysteamine, exhibits more favorable bioavailability and tolerability and has therefore progressed to clinical evaluation. The phase I CYTE-I-HD study evaluated escalating doses of cysteamine in nine patients and set the maximum tolerated dose between 10 and 50 mg/kg/day, with worsening movement and nausea as the dose-limiting effects and a hydrogen-sulphide odour on the breath once intake reached 40 mg/kg/day; around 20 mg/kg/day proved broadly tolerable (Dubinsky and Gray, 2006). A later controlled trial randomized 96 patients with early-stage HD to delayed-release cysteamine bitartrate (1,200 mg/day) or placebo for 18 months. The primary endpoint, change in UHDRS-TMS, did not differ significantly between groups (Verny et al., 2017). Thus, the trial did not demonstrate clinical efficacy, despite the preclinical neuroprotective effects reported for cystamine and cysteamine.

N-acetylcysteine (NAC) is a thiol-containing antioxidant that also serves as a cysteine precursor, thereby modulating oxidative stress through both direct redox effects and support of glutathione synthesis. The rationale for increasing cysteine availability in HD is further supported by the previously described reduction in CSE expression, which may limit cysteine availability in the striatum and increase susceptibility to oxidative and mitochondrial stress (Paul et al., 2014). Experimental studies suggest that the protective effects of NAC are primarily associated with modulation of redox homeostasis and mitochondrial function rather than with altered mHTT aggregation. In cells expressing huntingtin exon 1 containing expanded polyglutamine tracts, NAC reduced ROS production, nitric oxide levels, and protein oxidation, while restoring mitochondrial membrane potential. These effects were also observed in cells expressing constructs with 103 glutamine repeats, whereas neither the number nor the size of intracellular inclusions was significantly affected (Firdaus et al., 2006). Some antioxidant interventions have been reported to suppress basal autophagy in *Drosophila* and zebrafish HD models, raising the possibility that redox modulation may interact with autophagic pathways in a compound- and model-dependent manner (Underwood et al., 2010). This interaction should therefore be considered when interpreting antioxidant effects in experimental HD models. In the 3-NP rat model, NAC improved mitochondrial respiratory-chain activity, reduced oxidative damage and mitochondrial swelling, and was associated with improved motor performance and striatal histology (Sandhir et al., 2012). In the transgenic R6/1 line, chronic NAC delayed the onset and slowed the progression of motor deficits and reduced depressive-like behaviour. It did not restore the astrocytic glutamate-transporter (GLT-1) deficit, but it restored the striatum-specific loss of mitochondrial respiratory capacity and lowered mitochondrial oxidative damage (Wright et al., 2015). Adding NAC to a cysteine-enriched diet in R6/2 mice likewise delayed the motor phenotype, reduced brain atrophy, and extended survival (Paul et al., 2014). No completed clinical trial of NAC in HD has reported efficacy results. The ongoing phase II NAC-preHD study (NCT05509153) is evaluating NAC in premanifest *HTT*-expansion carriers using clinical and imaging outcomes over 3 years; no efficacy results are currently available.

α-Lipoic acid (α-LA) has been investigated as a modulator of thiol and GSH homeostasis. Upon reduction to dihydrolipoic acid, α-lipoic acid promotes the conversion of extracellular cystine to cysteine. The resulting cysteine can enter cells through neutral amino acid transporters, thereby providing an alternative route for increasing intracellular cysteine availability (Han et al., 1997). α-LA can also activate PI3K/Akt-dependent antioxidant signalling and induce phase II enzymes, including NQO1 and heme oxygenase 1, thereby protecting neuronal cells against glutathione depletion (Yamada et al., 2011). Together with its radical-scavenging and metal-chelating properties, these effects provide a mechanistic rationale for α-LA as an antioxidant. However, evidence for therapeutically relevant CNS exposure is limited, and adequate brain penetration in humans has not been established. HD-specific evidence also remains limited. α-LA increased survival in both the R6/2 and N171-82Q transgenic models, while a comparator antioxidant tested in the same work, the spin trap S-PBN did not significantly affect body weight or survival (Andreassen et al., 2001a). No trial has examined α-LA in HD.

Considered collectively, interventions targeting thiol and GSH metabolism have improved redox and mitochondrial measures in several HD models, but clinical evidence remains limited. Cysteamine did not meet its primary motor endpoint, and the other compounds in this class have not been evaluated clinically in HD. Moreover, human systemic exposure or tolerability data available for some of these compounds should not be equated with evidence of adequate brain penetration, regional CNS exposure, or biochemical target engagement in HD. Preclinical findings also suggest that antioxidant compounds may differ in their effects on autophagic pathways. The ongoing NAC-preHD study has not yet reported efficacy results and will provide prospective data on NAC treatment in the premanifest setting.

### 2.7 Lipid-membrane radical scavengers

In HD, oxidative damage is not confined to aqueous cellular compartments but also affects membrane lipids. ROS-mediated oxidation of polyunsaturated fatty acids initiates self-propagating lipid peroxidation reactions, generating reactive aldehyde products, including malondialdehyde (MDA), which can further modify proteins and nucleic acids. Lipophilic antioxidants that accumulate within biological membranes may interrupt these chain reactions by scavenging lipid-derived radicals and thereby limiting the propagation of membrane oxidative damage. This mechanism is distinct from that of water-soluble antioxidants and mitochondria-targeted agents discussed above. Among compounds acting through this mechanism, probucol has been evaluated in experimental models of HD.

Probucol is a lipophilic phenolic compound originally developed as a lipid-lowering drug. Its reported antioxidant effects include radical trapping, inhibition of lipid peroxidation, modulation of endogenous antioxidant enzymes and anti-inflammatory activity. *In vitro*, in rat striatal slices challenged with quinolinic acid, 3-NP, or both, probucol at 10 and 30 μM reduced ROS formation and lipid peroxidation across all three conditions, although it did not restore the mitochondrial dysfunction induced by 3-NP (Colle et al., 2012). A similar antioxidant effect was observed *in vivo*. Rats pretreated with probucol showed less weight loss and motor impairment and lower striatal lipid peroxidation following 3-NP exposure (Colle et al., 2013). The compensatory increases in SOD and catalase activity induced by 3-NP were also attenuated. In the absence of 3-NP exposure, probucol increased striatal GPx activity and reduced GSH content. The reduction in GSH content was attributed to activation of NRF2-dependent antioxidant signaling (Colle et al., 2013). The 3-NP-driven increase in inducible nitric oxide synthase (iNOS) was likewise suppressed by probucol (Colle et al., 2013). Because 3-NP is a toxin-induced model that reproduces mitochondrial and oxidative injury without reproducing the underlying *HTT* mutation, these findings primarily support pathway-specific antioxidant effects rather than disease-modifying activity in genetic HD. Probucol has also been evaluated in the full-length YAC128 transgenic model. In YAC128 mice given probucol (30 mg/kg daily) from postnatal day 30 for up to 5 months, treatment reduced depressive-like behaviour during early and mild symptomatic stages but did not improve rotarod performance. The antidepressant-like effect was not accompanied by increased hippocampal progenitor proliferation or neuronal differentiation (De Paula Nascimento-Castro et al., 2018). Although these studies demonstrate downstream biochemical and behavioural effects within the CNS, they did not establish regional brain exposure or a specific biochemical target-engagement marker for probucol.

No trial of probucol has been conducted in HD. Probucol has substantial prior clinical exposure as a lipid-lowering agent, but its use is limited by reductions in HDL cholesterol and prolongation of cardiac repolarization (Colle et al., 2013). This prior use provides information on human exposure and tolerability but does not establish safety at HD-relevant doses or durations, adequate CNS exposure, target engagement, or efficacy in HD.

### 2.8 Metal-binding agents

The final therapeutic class targets redox-active transition metals. Cu and Fe dyshomeostasis have been reported in HD, and interactions between these metals and HTT/mHTT may influence protein aggregation and redox chemistry. Fe and Cu can also promote ROS formation through Fenton and Fenton-like reactions (Cherny et al., 2012). The agents developed to modulate these processes include metal-protein attenuating compounds (MPACs), a family of 8-hydroxyquinoline derivatives that can redistribute metal ions across cell membranes rather than acting as classical systemic chelators (Summers et al., 2020a). Although some compounds in this class have physicochemical properties compatible with CNS exposure, evidence for brain penetration and regional target engagement should be considered separately from systemic exposure. Two have been studied in HD: clioquinol, the first-generation compound, and the second-generation derivative PBT2.

Clioquinol (5-chloro-7-iodo-8-hydroxyquinoline) is a bioavailable copper/zinc-binding compound that began as an antimicrobial (Li et al., 2010). Systemic use declined after an outbreak of subacute myelo-optic neuropathy in Japan; the mechanisms underlying this toxicity remain debated and have included proposed interactions with vitamin B_12_ metabolism. Interest revived once the compound was shown to lower amyloid burden in Alzheimer models by modulating copper and zinc availability, which helped establish the MPAC concept (Cherny et al., 2001). In HD, proof of principle came from PC12 cells expressing mHTT, in which clioquinol lowered accumulation of the mutant protein and the cell death that accompanies it, in a manner specific to polyglutamine length and independent of messenger-RNA level or protein-degradation rate (Nguyen et al., 2005). In R6/2 mice, oral clioquinol reduced mHTT aggregate burden and striatal atrophy, improved rotarod performance and body weight, modified glucose/insulin abnormalities, and extended survival (Nguyen et al., 2005). Clioquinol was not advanced to clinical testing in HD, whereas subsequent development focused on PBT2.

PBT2 (5,7-dichloro-2-((dimethylamino)methyl)-8-hydroxyquinoline) is a second-generation 8-hydroxyquinoline derivative developed with improved physicochemical and pharmacokinetic properties relative to clioquinol. Its compact and lipophilic structure has been proposed to favour CNS penetration, although physicochemical properties alone do not establish regional brain exposure or target engagement (Cherny et al., 2012). X-ray absorption spectroscopy shows PBT2 binding copper(II) in both 1:1 and 2:1 ligand-to-metal complexes. It can act as either a two- or a three-point ligand, providing flexibility for interactions with redox-active copper (Summers et al., 2020b). By modifying metal distribution, PBT2 may influence metal-dependent redox reactions and intracellular signaling. It also has a zinc-dependent action as a zinc ionophore that raises intracellular zinc and has been proposed to modulate intracellular calcium signaling through NMDA-receptor-related mechanisms. In the experimental systems studied, this was associated with attenuation of the calcium-dependent calpain–calcineurin pathway implicated in excitotoxic injury (Johanssen et al., 2015). In a *Caenorhabditis elegans* polyglutamine model PBT2 delayed and reduced paralysis, and in R6/2 mice daily oral dosing improved motor scores on the rotarod, shortened clasping, reduced striatal atrophy and lengthened median survival (Cherny et al., 2012). PBT2 is the only agent in this class to reach patients. The 26-week phase II REACH2HD trial randomised 109 patients with early- to mid-stage disease across three arms: placebo, 100 mg PBT2, or 250 mg daily (Angus et al., 2015). The drug met its primary endpoints of safety and tolerability, with adverse events (diarrhoea, headache, falls) mild and spread evenly across the groups. The principal secondary endpoint, a composite cognitive score, did not differ significantly from placebo at either dose, and motor outcomes were also unchanged. An exploratory improvement in an executive-function measure was reported in the 250-mg group, but this nominal secondary finding was not supported by improvement in the overall cognitive composite or motor outcomes (Angus et al., 2015). REACH2HD established short-term tolerability of PBT2 at the tested doses but did not demonstrate clinical efficacy. Moreover, the trial did not establish regional CNS exposure or biochemical target engagement in HD.

### 2.9 Emerging repurposing candidates

Several additional compounds are discussed separately as emerging repurposing candidates because their primary pharmacological actions extend beyond the core redox/mitochondrial framework or because HD-specific evidence remains less mature. Drug repurposing offers a route for evaluating related mechanisms using compounds with existing human exposure data. Bezafibrate, rosiglitazone, dichloroacetate, probucol, α-lipoic acid, and ebselen have prior human exposure in other indications. In addition, several approved agents or compounds in advanced clinical development target molecular pathways represented within the oxidative stress cascade in Figure 2 and may warrant further evaluation in HD.

Pridopidine provides a particularly relevant example. It is a selective agonist of the sigma-1 receptor (S1R), an endoplasmic-reticulum chaperone enriched at mitochondria-associated membranes (MAMs), which regulate ER–mitochondrial signaling. In YAC128 neurons, HD patient-derived neural stem cells, and patient lymphoblasts, pridopidine restored ER–mitochondrial contacts and improved antioxidant responses; when administered at an early disease stage *in vivo*, it also reduced mitochondrial ROS, normalised respiratory-complex activity, and delayed the onset of motor abnormalities (Naia et al., 2021). Its protective effects against mHTT-associated toxicity in primary striatal and cortical neurons and in iPSCs were abolished following loss of S1R, supporting a receptor-dependent mechanism (Eddings et al., 2019). S1R-dependent correction of dendritic spine loss and dysregulated ER calcium signalling has also been demonstrated in YAC128 co-culture models (Ryskamp et al., 2017), while a structurally distinct S1R agonist enhanced antioxidant capacity through NF-κB-associated signalling in mHTT-expressing neuronal cells (Hyrskyluoto et al., 2013). Collectively, these findings support S1R modulation as a mechanistically relevant therapeutic strategy rather than an effect restricted to pridopidine itself. Clinical findings, however, have been less conclusive. In PRIDE-HD, pridopidine did not produce a significant improvement in motor outcomes at 26 weeks across the four evaluated doses (Reilmann et al., 2019). Similarly, the phase III PROOF-HD trial did not meet its primary endpoint of change in TFC at week 65 (least-squares mean difference −0.18, 95% CI −0.49 to 0.14; p = 0.26), nor its key secondary composite UHDRS endpoint (Reilmann et al., 2025). A prespecified sensitivity analysis restricted to participants not exposed to antidopaminergic medications showed numerical differences favouring pridopidine across several clinical measures (Reilmann et al., 2025). These subgroup findings should be considered exploratory and do not alter the negative primary trial result.

Metformin, dimethyl fumarate, and edaravone have more preliminary HD-specific evidence. Metformin activates AMPK, rescues mHTT-expressing cells from toxicity, preserves mitochondrial membrane potential, limits excess fission (Jin et al., 2016). In a cross-sectional observational analysis of the Enroll-HD cohort, metformin use was associated with better cognitive performance in patients (Hervás et al., 2017). However, this association cannot be interpreted as evidence of treatment efficacy because of potential confounding by indication, comorbidities, concomitant medications, and other differences between treated and untreated participants. Dimethyl fumarate, licensed for multiple sclerosis, activates NRF2 by S-alkylating Keap1, providing a mechanistic link to the NRF2/ARE pathway discussed above, and its metabolite monomethyl fumarate does so without the glutathione depletion that dimethyl fumarate itself causes (Ahuja et al., 2016). HD-specific therapeutic efficacy has not been established. Edaravone, a licensed radical scavenger, blocked the mutant-cell-specific rise in mitochondrial ROS in STHdhQ111 striatal cells (Okada et al., 2021). Thus, the available HD evidence for edaravone remains limited to preclinical cellular studies.

## 3 Discussion and future directions

The evidence summarized in Tables 1, 2 highlights a substantial translational gap between preclinical and clinical studies. Most compounds produced beneficial effects in cellular or animal models, whereas clinical efficacy has remained limited. Most have not yet been evaluated in individuals with HD, and among those that progressed to controlled clinical testing, none has demonstrated efficacy on a prespecified primary efficacy endpoint.

**TABLE 1:** Preclinical evidence for pharmacological agents targeting oxidative stress in Huntington’s disease**.**

Class/mechanism	Compound	Evidence level	Model	Dose/exposure	Major outcome	Principal preclinical limitation	References
PPAR agonists	Rosiglitazone	*In vitro* HD-specific knock-in cell model	STHdhQ111 striatal cells; thapsigargin challenge	20 µM; 24 h	Reduced ROS and loss of mitochondrial membrane potential; increased mitochondrial mass	Genetic cell model combined with acute pharmacological stress	Quintanilla et al., 2008
	​	*In vitro* engineered cellular HD model	mHTT-expressing N2A cells	10 µM; 48 h	Increased PPARγ/PGC-1α/NRF1/TFAM signaling; improved mitochondrial biogenesis, proteostasis and survival	Engineered neuroblastoma model; not patient-derived	Chiang et al., 2015
	​	*In vivo* fragment-based transgenic HD model	R6/1 mice	10 mg/kg body weight dose; IP daily from 21 weeks of age for 6 weeks	Improved grip strength, cardiac function and peripheral bioenergetic indices	Effects were predominantly peripheral; fragment-based model	Tomczyk et al., 2022
	​	*In vivo* fragment-based transgenic HD model	R6/2 mice	0.01% in diet from 4 weeks of age for 8 weeks	Improved body weight and motor performance; reduced aggregates; prolonged survival	Rapid fragment-based model; variable dietary exposure with disease progression; no prevention of brain atrophy	Chiang et al., 2010
PPAR agonists	Bezafibrate	*In vivo* fragment-based transgenic HD model	R6/2 mice	0.5% in diet from 5 weeks of age; chronic treatment	Restored PPAR/PGC-1α signaling; improved phenotype, mitochondrial measures and survival	Rapid fragment-based model; early treatment and systemic pan-PPAR effects limit CNS-specific interpretation	Johri et al., 2012
	​	*In vivo* full-length transgenic HD model	BACHD mice	0.5% in diet from 5 weeks of age; chronic treatment	Increased mitochondrial biogenesis and antioxidant capacity; attenuated motor/pathological phenotype	Full-length transgenic model, but early chronic treatment and non-selective pan-PPAR activation limit attribution to a specific receptor or CNS mechanism	Chandra et al., 2016
	​	*In vivo* toxin-induced HD-like model	3-NP rats	75 mg/kg/day by oral gavage for 7 days, starting concomitantly with 3-NP administration	Preserved antioxidant, mitochondrial and synaptic measures	Acute toxin-induced model that does not reproduce the *HTT* mutation; concomitant treatment with bezafibrate and 3-NP limits assessment of efficacy after disease onset	Brondani et al., 2023
PPAR agonists	KD3010	*In vitro* human patient-derived HD model	iPSC-derived medium spiny-like neurons	1 µM; 48 h	Reduced mHTT-related toxicity	Patient-derived cells lack systemic and longitudinal disease context	Dickey et al., 2016
	​	*In vivo* fragment-based transgenic HD model	N171-82Q mice	50 mg/kg/day by IP, starting at 6 weeks of age, chronic treatment	Improved motor function, delayed neurodegeneration and prolonged survival	Fragment-based model with relatively rapid disease progression; treatment was initiated early, limiting assessment of efficacy after established symptomatic disease	Dickey et al., 2016
NRF2/ARE activators	CDDO-EA/CDDO-TFEA	*In vivo* fragment-based transgenic HD model	N171-82Q mice	CDDO-EA: 100 or 200 mg/kg diet; CDDO-TFEA: 100, 200, or 400 mg/kg diet, starting at 30 days of age and continued chronically	Increased ARE-dependent gene expression in brain/periphery; reduced oxidative damage; improved motor phenotype and survival	Early presymptomatic treatment in a rapidly progressive fragment-based model; dietary dosing produced different and relatively low brain exposure, particularly for CDDO-EA	Stack et al., 2010
Quinone analogues	CoQ10	*In vivo* full-length knock-in HD model	CAG140 mice	0.2% or 0.6% in diet for 9 weeks, starting at 8 weeks of age	0.2% improved early behavioural and transcriptional abnormalities; aggregate burden unchanged	Early intervention in a slowly progressive knock-in model; non-monotonic dose response and no reduction in huntingtin aggregate burden	Hickey et al., 2012
Bioenergetic substrates	Creatine	*In vitro* neuronal toxin/stress model	Cultured striatal and hippocampal neurons exposed to glutamate or 3-NP	3 mM or 10 mM; 2–5 days	Reduced toxin/excitotoxic injury	Non-genetic stress model; inability of creatine to directly inhibit the mitochondrial permeability transition pore under physiological conditions	Brustovetsky et al., 2001
	​	*In vivo* fragment-based transgenic HD model	R6/2 mice	2% creatine in diet +1% CoQ10; from 5 weeks of age; chronically	Combination improved survival, rotarod performance and oxidative measures	Combination prevents attribution of the effect to creatine alone	Yang et al., 2009
Bioenergetic substrates	DCA	*In vitro* HD-specific knock-in cell model	STHdhQ111 cells	300 μM, 1 mM, and 3 mM; 24 h	Increased mitochondrial respiration and reduced ROS	Cellular evidence only	Naia et al., 2017
	​	*In vivo* fragment-based transgenic HD models	R6/2 and N171-82Q mice	100 mg/kg/day in drinking water from 4 weeks of age; chronic treatment	Improved survival/motor phenotype and corrected PDHC-related abnormalities	Rapid fragment models	Andreassen et al., 2001b
Bioenergetic substrates	Nicotinamide	*In vivo* fragment-based transgenic HD model	R6/1 mice	250 mg/kg/day in drinking water/osmotic pump; treatment from 8 to 20 weeks of age	Improved motor phenotype; increased BDNF and PGC-1α expression	Fragment-based model; early intervention; NAD^+^ target engagement not directly demonstrated	Hathorn et al., 2011
Mitochondria-targeted antioxidants	MitoQ	*In vitro* HD-specific knock-in cell model	STHdhQ111/Q111 cells	25 nM; 24 h treatment	Reduced ROS/lipid peroxidation; increased ATP and mitochondrial/synaptic transcripts	Cell-based model; short exposure	Yin et al., 2016
	​	*In vivo* fragment-based transgenic HD model	R6/2 mice	500 μM in drinking water, *ad libitum*, from 5 to 11 weeks of age	Improved motor performance and oxidative measures, mainly in skeletal muscle	Rapid fragment-based R6/2 model; early presymptomatic treatment; limited brain exposure with predominantly peripheral effects	Pinho et al., 2020
Mitochondria-targeted antioxidants	XJB-5-131	*In vitro* primary genetic neuronal HD model	Primary striatal neurons from embryonic day 17 HD150KI (HdhQ150 mice)	1 µM for 7 days	Mitochondrial localization; improved survival of primary striatal neurons	*In vitro* genetic neuronal model; no systemic PK or brain-exposure assessment	Xun et al., 2012
	​	*In vivo* full-length knock-in HD model	HD150KI/HdhQ150 mice	1 mg/kg IP, 3×/week, from 7 to 57 weeks of age	Reduced weight loss and motor decline; improved grip strength; reduced mtDNA oxidative damage; restored mtDNA abundance; improved mitochondrial respiratory reserve capacity	Long-term parenteral dosing; mouse knock-in model	Xun et al., 2012
	​	*In vivo* full-length knock-in HD model	HdhQ150 mice	2 mg/kg IP, 3×/week; initiated in older symptomatic mice	Attenuated several established pathological and behavioural abnormalities	Late-stage intervention in a mouse model; parenteral dosing; no human CNS exposure /Target-engagement data	Polyzos et al., 2016
Mitochondria-targeted antioxidants	SS-31/elamipretide	*In vitro* HD-specific knock-in cell model	STHdhQ111/Q111 cells	25 nM; 24 h	Reduced ROS/lipid peroxidation; increased ATP and mitochondrial/synaptic markers	HD-specific evidence limited to cell model	Yin et al., 2016
Glutathione/cysteine donors	Ebselen	*In vitro* fragment-based mammalian HD cell model	Neuron-like PC12 cells inducibly expressing Htt103Q	10 μM; single treatment during 72 h Htt103Q induction	Reduction in caspase-3/7 activation; reduced mHTT associated ROS; did not inhibit autophagy	Fragment-expression cell model	Mason et al., 2013
	​	*In vivo* fragment-based *Drosophila* HD model	*Drosophila* expressing Htt93Qex1 pan-neuronally	100 or 300 μM in food for 7 days after eclosion	Reduced photoreceptor neurodegeneration	Non-mammalian fragment-based model	Mason et al., 2013
Glutathione/cysteine donors	Cystamine/cysteamine	*In vitro* HD-specific knock-in cell model + toxin challenge	STHdhQ111 cells + 3-NP	Cystamine: 50 and 250 μM; cysteamine: 50–500 μM; 16 h	Preserved mitochondrial membrane potential and GSH; protection was lost after GSH depletion	Genetic HD cell model combined with acute toxin challenge; does not reproduce chronic disease progression	Mao et al., 2006
	​	*In vivo* fragment-based transgenic HD model	R6/2 mice	112–225 mg/kg/day IP from postnatal day 21 to ∼17 weeks; 225 mg/kg/day orally in drinking water (900 mg/L) in a prenatal/postnatal paradigm	Improved survival, weight and motor phenotype	Rapid fragment-based model; early treatment; chronic daily parenteral dosing	Dedeoglu et al., 2002
	​	*In vivo* full-length transgenic HD model	YAC128 mice	225 mg/kg/day in drinking water (900 mg/L); 4–12 or 7–12 months of age	Prevented striatal neuronal loss and atrophy	Structural preservation was not accompanied by comparable improvement in motor dysfunction or DARPP-32 expression	Van Raamsdonk et al., 2005
Glutathione/cysteine donors	NAC	*In vitro* engineered cellular HD model	COS-7 cells expressing httEx1-72Q/103Q; neuronal SK-N-SH and PC12 httEx1-polyQ models	2 mM for 12 h	Reduced ROS/HE oxidation at huntingtin inclusion bodies; reduced local cellular/morphological damage around inclusions; inclusion size/number largely unchanged	Artificial fragment-expression system; high *in vitro* concentration; short exposure; no intact CNS context	Firdaus et al., 2006
	​	*In vivo* toxin-induced HD-like model	3-NP rats	100 mg/kg/day by gavage for 12 days; administered 1 h before 3-NP (25 mg/kg IP for 4 days)	Improved mitochondrial respiratory function and redox status; reduced apoptotic signaling; improved motor/cognitive and histological outcomes	Toxin-induced model; does not reproduce the *HTT* mutation or chronic genetic disease course; short prophylactic/early-treatment paradigm	Sandhir et al., 2012
	​	*In vivo* fragment-based transgenic HD model	R6/1 mice	500 mg/kg/day IP, once daily from 8 to 17 weeks of age	Delayed motor deficits; reduced striatal mitochondrial oxidative damage; restored mitochondrial respiratory capacity	High-dose daily parenteral regimen; fragment-based model; treatment began around onset of motor deficits; no direct CNS exposure/target-engagement measurement	Wright et al., 2015
	​	*In vivo* fragment-based transgenic HD model	R6/2 mice	0.8% cystine diet +20 mM NAC in drinking water from 4 weeks of age	Delayed hindlimb clasping; improved rotarod performance and grip strength; partially preserved brain mass and striatal volume; prolonged survival	Rapid fragment-based R6/2 model; combined cysteine + NAC intervention prevents attribution of benefit specifically to NAC	Paul et al., 2014
Glutathione/cysteine donors	α-Lipoic acid	*In vivo* fragment-based transgenic HD models	R6/2 and N171-82Q mice	100 mg/kg/day, starting at 4 weeks of age	Prolonged survival in both HD models; delayed weight loss in N171-82Q mice	Fragment-based rapidly progressive models; early treatment; limited mechanistic and CNS target-engagement assessment	Andreassen et al., 2001a
Lipid-membrane radical scavengers	Probucol	*Ex vivo* toxin-based striatal model	Rat striatal slices + 3NP/quinolinic acid (QA)	10 or 30 μM; co-incubated with toxins for 2 h, then re-added during an additional 4 h incubation; QA 1 mM, 3-NP 1 mM, or QA + 3-NP 0.1 mM each	Prevented ROS formation and lipid peroxidation in all toxin conditions; preserved mitochondrial function in QA and QA + 3-NP, but not in 3-NP alone	Acute *ex vivo* toxin model; does not reproduce the *HTT* mutation; antioxidant protection did not reverse primary 3-NP-induced mitochondrial dysfunction	Colle et al., 2012
	​	*In vivo* toxin-induced model	3-NP rats	3.5 mg/kg/day in drinking water for 2 months, followed by 3-NP 25 mg/kg IP once daily for 6 days	Reduced weight loss and motor impairment; lowered striatal lipid peroxidation and iNOS induction; attenuated SOD/catalase responses; increased striatal GPx activity	Toxin model does not reproduce the *HTT* mutation; probucol did not reverse 3-NP-induced complex II inhibition	Colle et al., 2013
	​	*In vivo* full-length transgenic HD model	YAC128 mice	30 mg/kg/day from postnatal day 30 for up to 5 months	Reduced depressive-like behaviour during early/mild symptomatic stages; no rotarod benefit; no increase in hippocampal progenitor proliferation or neuronal differentiation	Narrow behavioural effect without broader motor or neurogenic rescue; no regional brain exposure or specific target-engagement measure	De Paula Nascimento-Castro et al., 2018
Metal-binding agents	PBT2	*In vivo* non-mammalian polyQ model	*C. elegans* AM140 expressing Q35-YFP in body-wall muscle	10 μg/mL; exposure initiated at L4 stage for 24 h and continued in young adults	Delayed polyQ-associated paralysis; benefit occurred without a clear reduction in visible polyQ-YFP aggregation	Non-mammalian polyQ overexpression model; polyQ expressed in muscle rather than neurons	Cherny et al., 2012
	​	*In vivo* fragment-based transgenic HD model	R6/2 mice	30 mg/kg/day by oral gavage, starting at 3 weeks of age and continued throughout the study/lifespan	Improved rotarod/clasping and reduced striatal pathology	Rapid fragment-based model; treatment initiated before overt motor symptoms; mechanism/metal target engagement was not directly assessed	Cherny et al., 2012
Metal-binding agents	Clioquinol	*In vitro* engineered cellular HD model	PC12 and HEK293 cells expressing Htt exon 1 with expanded polyQ (Q103)	0.5–8 μM; cells exposed for 30 min before and 30 min after transfection; outcomes assessed up to 48 h	Reduced mHTT protein accumulation and cell death in a dose-dependent manner; no significant effect on *HTT* mRNA abundance or protein degradation rate	Artificial fragment-expression system; short-term exposure; effective and cytotoxic concentration ranges overlap at higher doses	Nguyen et al., 2005
	​	*In vivo* fragment-based transgenic HD model	R6/2 mice	30 mg/kg/day by oral gavage, from ∼3 weeks of age	Reduced aggregated HTT accumulation, striatal atrophy and clasping; improved rotarod performance and weight trajectory; normalized glucose/insulin abnormalities; increased mean lifespan	Rapid fragment-based model; treatment initiated very early; chronic gavage; mechanism not shown to depend specifically on metal chelation	Nguyen et al., 2005

Summary of experimental models, doses, major outcomes, and key limitations. Abbreviations: 3-NP, 3-nitropropionic acid; AMPK, AMP-activated protein kinase; ARE, antioxidant response element; ATP, adenosine triphosphate; BACHD, bacterial artificial chromosome Huntington disease mouse model; BDNF, brain-derived neurotrophic factor; CDDO-EA, CDDO-ethyl amide; CDDO-TFEA, CDDO-trifluoroethyl amide; CNS, central nervous system; CoQ10, coenzyme Q10; CSE, cystathionine γ-lyase; DCA, dichloroacetate; GPx, glutathione peroxidase; GSH, reduced glutathione; HD, huntington disease; iNOS, inducible nitric oxide synthase; IP, intraperitoneal; iPSC, induced pluripotent stem cell; mHTT, mutant huntingtin; mtDNA, mitochondrial DNA; NAC, N-acetylcysteine; NAD^+^, nicotinamide adenine dinucleotide; NO, nitric oxide; NQO1, NAD(P)H:quinone oxidoreductase 1; NR, nicotinamide riboside; NRF1, nuclear respiratory factor 1; NRF2, nuclear factor erythroid 2-related factor 2; PDHC, pyruvate dehydrogenase complex; PGC-1α, peroxisome proliferator-activated receptor gamma coactivator 1-alpha; polyQ, polyglutamine; PPAR, peroxisome proliferator-activated receptor; QA, quinolinic acid; ROS, reactive oxygen species; SOD, superoxide dismutase; TFAM, mitochondrial transcription factor A.

**TABLE 2:** Clinical evidence for pharmacological agents targeting oxidative stress-related pathways in Huntington’s disease**.**

Class/mechanism	Compound	Study design/population	Dose/duration	Major outcome	Clinical interpretation	Principal translational limitation	References
Quinone analogues	CoQ10	CARE-HD; randomized controlled trial; early-stage HD	600 mg/day; 30 months	No significant effect on TFC primary outcome	No demonstrated clinical efficacy	CNS/striatal target engagement not demonstrated	Huntington Study Group, 2001
	​	2CARE; randomized controlled trial; early-stage HD	2,400 mg/day; long-term	No benefit on TFC or secondary outcomes; trial stopped for futility	Negative efficacy result	Limited CNS exposure and lack of demonstrated striatal target engagement	McGarry et al., 2017
Quinone analogues	Idebenone	Randomized placebo-controlled trial; HD patients	270 mg/day 1 year	No significant benefit on primary clinical outcomes	No demonstrated clinical efficacy	Neuronal bioactivation may be limited by low NQO1 expression	Ranen et al., 1996
Bioenergetic substrates	Creatine	PRECREST; premanifest carriers/individuals at genetic risk	30 g/day; 18 months	Exploratory MRI differences in cortical and striatal atrophy	Exploratory biomarker findings only	Imaging outcome; not powered to establish clinical efficacy	Rosas et al., 2014
	​	CREST-E; symptomatic HD	Up to 40 g/day; long-term treatment	Trial terminated for futility	Negative efficacy result	No demonstrated functional benefit	Hersch et al., 2017
Bioenergetic substrates	Triheptanoin	Clinical study in early-stage HD	1 g/kg/day; up to 12 months	No effect on prespecified 6-month caudate-atrophy endpoint; *post hoc* 12-month comparison suggested less atrophy	Primary endpoint negative; *post hoc* finding exploratory	External comparator and *post hoc* analysis	Mochel et al., 2025
Glutathione/cysteine donors	Cysteamine	Randomized clinical study; early-stage HD	1,200 mg/day 18 months	Numerically smaller deterioration, but between-group difference not significant	No demonstrated clinical efficacy	Primary comparison not statistically significant	Verny et al., 2017
Glutathione/cysteine donors	NAC	Phase II randomized, quadruple-masked, placebo-controlled; premanifest *HTT*-expansion carriers; planned n = 160; recruiting	1 g orally twice daily; up to 3 years	Primary endpoints: caudate atrophy rate on MRI and motor phenoconversion; no efficacy results available	Ongoing trial	Premanifest population; long follow-up required; clinical relevance of imaging and phenoconversion endpoints remains to be established	ClinicalTrials.gov, NCT05509153; accessed 23 September 2026
Bioenergetic substrates	NR	Phase II randomized, quadruple-masked, placebo-controlled; adults aged 18–80 years with manifest early- to mid-stage HD; planned n = 120; recruiting	2000 mg/day orally for 2 years	Primary endpoint: change from baseline in composite UHDRS (cUHDRS) at 730 days; no efficacy results available	Ongoing trial	Results will apply to manifest early- to mid-stage HD; long treatment duration and multimodal secondary biomarker outcomes; clinical benefit remains unestablished	ClinicalTrials.gov, NCT06853743; accessed 23 September 2026
Metal-binding agents	PBT2	REACH2HD; phase II; early- to mid-stage HD	100 or 250 mg/day; 26 weeks	No effect on cognitive composite or motor scale; exploratory signal on Trail Making part B	Efficacy not established	Secondary/exploratory signal only	Angus et al., 2015

Summary of study design, dosing, principal outcomes, clinical interpretation, and key translational limitations. Abbreviations: ADL, activities of daily living; CNS, central nervous system; CoQ10, coenzyme Q10; HD, Huntington disease; MRI, magnetic resonance imaging; NAC, N-acetylcysteine; NAD^+^, nicotinamide adenine dinucleotide; NQO1, NAD(P)H:quinone oxidoreductase 1; NR, nicotinamide riboside; QNE, quantified neurologic examination; TFC, total functional capacity; UHDRS, Unified Huntington’s Disease Rating Scale; UHDRS-TMS, Unified Huntington’s Disease Rating Scale–Total Motor Score.

Several factors may contribute to the discrepancy between preclinical and clinical findings. One is insufficient exposure at the relevant target site, particularly within the CNS. When assessing translational potential, systemic exposure, brain penetration, regional CNS exposure, biochemical target engagement, downstream pharmacodynamic effects, and clinical efficacy should be considered separately, because evidence at one level does not necessarily imply evidence at the next. For example, the absence of evidence for striatal target engagement complicates interpretation of the negative 2CARE result (CoQ10 supplementation) and leaves open whether limited CNS exposure contributed to therapeutic failure (McGarry et al., 2017). Idebenone additionally requires two-electron reduction by NQO1 for efficient antioxidant activity. Because neuronal NQO1 expression is relatively low, cell-type-dependent bioactivation may limit its activity in neurons (Jaber et al., 2020). MitoQ was developed specifically to improve mitochondrial delivery, yet in R6/2 mice its beneficial effects were largely confined to skeletal muscle, while brain autophagy markers remained unchanged, consistent with limited brain exposure in this model (Pinho et al., 2020). In addition, 1 year of oral MitoQ treatment in Parkinson’s disease did not slow clinical progression on the measured outcomes (Snow et al., 2010). For these compounds, negative or limited efficacy therefore cannot be readily separated from inadequate CNS exposure or insufficient target engagement.

Moreover, these contrasting results raise the possibility that disease stage influences therapeutic responsiveness, although differences between the trials prevent causal attribution to timing alone. Creatine at 30 g/day was associated with exploratory differences in cortical and striatal atrophy on serial MRI at six and 18 months (Rosas et al., 2014). In symptomatic patients, the CREST-E trial of creatine doses up to 40 g/day was terminated for futility, with functional decline numerically greater in the treatment group than in the placebo group (Hersch et al., 2017). Triheptanoin did not affect the prespecified 6-month caudate-atrophy endpoint, whereas a *post hoc* comparison with an external control cohort suggested less atrophy at 12 months (Mochel et al., 2025). After 18 months of treatment in early-stage HD, cysteamine did not produce a statistically significant between-group difference in the primary clinical outcome (Verny et al., 2017). Two ongoing trials may provide further information on whether intervention before or early in clinical manifestation is associated with measurable biological effects: NAC-preHD (NCT05509153) in premanifest expansion carriers, and NAD-HD (NCT06853743) in early-stage patients. These ongoing studies will provide prospective data on biological and clinical effects of redox-directed interventions in premanifest and early-stage HD populations.

A third possibility is that treatment effects may occur in domains not captured by the primary endpoint. In YAC128 mice, cystamine preserved striatal volume and neuron number yet left motor performance and DARPP-32 unchanged (Van Raamsdonk et al., 2005). Probucol reduced depressive-like behaviour in the same model without improving rotarod performance (De Paula Nascimento-Castro et al., 2018). In REACH2HD, an exploratory nominal improvement was reported for Trail Making Part B, an executive-function measure, whereas the cognitive composite and motor outcomes were unchanged (Angus et al., 2015). Redox-directed interventions may affect biological and clinical domains differently. Clinical trials have frequently relied on motor, functional or composite clinical outcomes that may not capture all biological effects suggested by preclinical studies. Future studies may benefit from aligning prespecified endpoints with the expected mechanism of action and disease stage, while incorporating validated structural, metabolic or domain-specific measures where appropriate.

The pharmacological strategies reviewed here have generally shown limited effects on mHTT aggregation itself. In CAG140 mice, CoQ10 treatment did not alter huntingtin aggregate burden (Hickey et al., 2012) while NAC had no detectable effect on inclusion number or size, even in models carrying 103 CAG repeats (Firdaus et al., 2006). Similarly, nicotinamide improved motor performance in R6/1 mice without a corresponding reduction in aggregate pathology (Hathorn et al., 2011). These observations are consistent with predominantly downstream effects on redox and metabolic pathways rather than a consistent reduction in mHTT aggregation. In this context, XJB-5–131 is of particular interest because its effects may extend beyond the attenuation of downstream oxidative damage. Somatic CAG expansion is strongly influenced by DNA-maintenance pathways, particularly mismatch-repair factors. Oxidative DNA lesions may also contribute to repeat instability through DNA-repair-associated mechanisms, although the magnitude of this contribution in human HD remains uncertain. In HdhQ150 mice, XJB-5–131 reduced both oxidative DNA damage and somatic CAG expansion (Xun et al., 2012). These parallel effects do not establish that reduced oxidative DNA damage directly mediates the reduction in somatic CAG expansion, but they support further investigation of the relationship between redox state, DNA damage, and repeat instability. This is of particular interest given the growing genetic and experimental evidence identifying somatic repeat expansion as an important modifier of HD onset and progression (Budworth et al., 2015). Further studies are needed to determine whether reducing oxidative DNA damage in vulnerable neuronal populations can influence somatic repeat instability or other disease-relevant outcomes.

The available data also support several testable hypotheses for combination therapy. One theoretical approach would combine idebenone with an NRF2 activator, because NQO1 is an NRF2-regulated enzyme required for idebenone bioactivation (Stack et al., 2010; Jaber et al., 2020). The only combination discussed here with direct *in vivo* HD data is creatine plus CoQ10, which produced somewhat greater survival and motor effects than either treatment alone in R6/2 mice (Yang et al., 2009). Pairing a redox agent with a huntingtin-lowering therapy represents another mechanistically distinct possibility. Thiol antioxidants suppress basal autophagy in fly and zebrafish HD models and can attenuate the beneficial effects of autophagy-inducing drugs (Underwood et al., 2010), whereas raising GPx activity relieves oxidative stress without that liability (Mason et al., 2013). Combining a thiol donor with a strategy that depends on autophagic clearance of mHTT could result in pharmacodynamic antagonism. With the exception of creatine plus CoQ10, these combination strategies remain mechanistic hypotheses, and the reported autophagy-related interactions are specific to the compounds and experimental models studied and should not be generalized to broader drug classes or therapeutic approaches.

Future redox-directed trials should aim to identify patients most likely to benefit from therapies targeting oxidative stress by assessing baseline redox status together with relevant comorbidities, concomitant medications, and disease stage. Where feasible, these parameters should also be monitored longitudinally as potential measures of target engagement and treatment response. Redox-related abnormalities have been reported in peripheral biofluids before motor onset, although their reproducibility and clinical utility remain to be established (Klepac et al., 2007; Tang et al., 2020). Somatic CAG expansion has emerged as an important modifier of HD onset and progression, supported by genetic studies implicating multiple DNA-maintenance genes (Lee et al., 2015, 2019). Future studies could therefore explore biomarker-based stratification using validated measures of redox imbalance and, where feasible, molecular or genetic markers associated with somatic expansion propensity. Such approaches would require prospective validation before being adopted as eligibility criteria. Future stratification approaches may also consider concomitant medication and disease stage, although the available evidence supporting these factors remains exploratory.

Several agents reviewed here show prominent peripheral effects despite limited or uncertain CNS activity. The beneficial effects of MitoQ in R6/2 mice were largely confined to skeletal muscle (Pinho et al., 2020); rosiglitazone corrected cardiac and skeletal-muscle energetics in R6/1 mice (Tomczyk et al., 2022); bezafibrate reversed the oxidative-to-glycolytic fibre shift and the brown-adipose vacuolation of R6/2 mice (Johri et al., 2012); elamipretide received accelerated FDA approval for Barth syndrome, providing human exposure and tolerability data in a non-HD population (U.S. Food and Drug Administration, 2026). Muscle wasting, cardiomyopathy and impaired glucose handling may independently contribute to disability in HD. Peripheral target engagement may therefore remain clinically relevant even when CNS exposure is limited, particularly for interventions directed at muscle or cardiac phenotypes. Future trials could incorporate prespecified peripheral outcomes, including muscle strength, cardiac measures, body composition and metabolic biomarkers, where these correspond to the expected mechanism of action.

Several limitations constrain the interpretation of the available evidence. Considerable heterogeneity in experimental models, dosing regimens, treatment duration, and outcome measures precludes meaningful quantitative synthesis; consequently, comparisons across studies are necessarily qualitative. A substantial proportion of the evidence also derives from rapidly progressive fragment-based transgenic models, including R6/2 and N171-82Q mice. Although these models are valuable for mechanistic and proof-of-concept studies, their accelerated disease course and expression of N-terminal mHTT fragments may not fully recapitulate the slowly progressive, decades-long pathology of human HD and may therefore limit the translational relevance of therapeutic effects observed over relatively short treatment periods (Crook and Housman, 2011; Pouladi et al., 2013). Their rapid phenotype may also favour detection of effects on fragment toxicity and aggregate-related pathology that are less prominent in the human disease. Toxin-based models, such as 3-nitropropionic acid and quinolinic acid models, reproduce several features relevant to HD, including striatal injury, mitochondrial dysfunction, excitotoxicity, and oxidative stress, but they do not reproduce the underlying *HTT* mutation or the lifelong genetic background of the disease (Browne, 2008). They are therefore useful mainly for studying specific pathogenic mechanisms. Full-length transgenic and knock-in models, including BACHD, YAC128, CAG140, and HdhQ150 mice, better preserve the genetic context of HD and usually develop a slower phenotype. However, behavioural and neuropathological changes may be milder or delayed, which often requires longer experiments (Menalled, 2005; Pouladi et al., 2013). For this reason, results reproduced across more than one model may be more informative than findings obtained in a single experimental system. Additional limitations include frequent initiation of treatment before substantial disease expression in animals and the potential for publication bias toward positive preclinical findings.

## 4 Conclusion

Oxidative stress in HD is closely linked to mitochondrial dysfunction, impaired redox homeostasis, and oxidative DNA damage, and may also interact with pathways influencing somatic CAG instability. These pathways provide a substantial mechanistic basis for therapeutic investigation; however, preclinical activity has not translated into convincing clinical benefit. The available clinical data indicate that future studies will need to demonstrate adequate target-site exposure and biochemical target engagement, while accounting for disease stage and selecting outcomes appropriate to the proposed mechanism. Because most redox-directed interventions do not directly lower mHTT, combining them with therapies acting on upstream disease mechanisms remains a mechanistic hypothesis that requires direct experimental and clinical evaluation.
